# Sensitivity of Dendritic Cells to Microenvironment Signals

**DOI:** 10.1155/2016/4753607

**Published:** 2016-03-21

**Authors:** Juliana Maria Motta, Vivian Mary Rumjanek

**Affiliations:** Laboratório de Imunologia Tumoral, Instituto de Bioquímica Médica Leopoldo de Meis, Universidade Federal do Rio de Janeiro, 21941-902 Rio de Janeiro, RJ, Brazil

## Abstract

Dendritic cells are antigen-presenting cells capable of either activating the immune response or inducing and maintaining immune tolerance. They do this by integrating stimuli from the environment and changing their functional status as a result of plasticity. The modifications suffered by these cells have consequences in the way the organism may respond. In the present work two opposing situations known to affect dendritic cells are analyzed: tumor growth, leading to a microenvironment that favors the induction of a tolerogenic profile, and organ transplantation, which leads to a proinflammatory profile. Lessons learned from these situations may help to understand the mechanisms of modulation resulting not only from the above circumstances, but also from other pathologies.

## 1. Introduction

Although studies of the immune system mainly focus on its role in protecting against infections, this is only part of its function in helping to maintain the homeostasis of the organism. The tolerance and unresponsiveness to self-antigens, as well as the ability to terminate immune responses after pathogens control, are mechanisms carefully regulated and essential to keep and return to the steady-state. To be able to maintain the necessary equilibrium, the system must adapt to different challenges producing distinct and sometimes paradoxical responses.

Dendritic cells contribute to this purpose exhibiting a large spectrum of phenotypes and activities. The present review examines the role played by dendritic cells in two extremes and opposing situations (tumor microenvironment versus organ transplantation) where the plasticity of these cells is clearly observed and is directly related to their microenvironment.

## 2. Dendritic Cell Origin and Function

Dendritic cells are cells specialized in antigen presentation. These cells are capable of perceiving environment imbalances, capturing self and non-self-antigens, and processing and presenting them as peptides linked to the major histocompatibility complexes (MHC) to T lymphocytes. Dendritic cells are extremely sensitive to microenvironment signals and they scan the organism, especially the sites where there is more probability of antigen entrance. In other words, dendritic cells efficiently instruct the adaptive immune system in response to peripheral cues, as discussed by Merad et al. [1].

Evidence suggests that dendritic cells are originated from both myeloid and lymphoid hematopoietic progenitors. The cytokine Flt3 ligand (Flt3L) was shown to be necessary for dendritic cell development in the bone marrow of both human and mice. Furthermore, this cytokine plays a role later in murine and human lymphoid organs. Deficiency of its receptor (Flt3) is associated with these cells depletion in mice [2–5].* In vivo*, it was demonstrated that Flt3L administration can drive and expand dendritic cell generation along both the lymphoid and myeloid developmental pathways from Flt3^+^ progenitor cells [3].

In humans, dendritic cells represent only 0.1–0.5% of mononuclear cells present in peripheral blood [6]. Due to the low concentration and difficulty in obtaining these cells, the work with human dendritic cells was limited for years. However, obtaining human dendritic cells,* in vitro*, from bone marrow precursors or monocyte-induced differentiation has made it possible to study the biology of these cells [7, 8]. Granulocyte-macrophage colony-stimulating factor (GM-CSF) combined with tumor necrosis factor-alpha (TNF-*α*) is efficient in differentiating bone marrow CD34^+^ cells into dendritic cells [7, 9], while the best mixture to differentiate monocytes into dendritic cells is interleukin-4 (IL-4) plus GM-CSF [8]. In this context, GM-CSF was characterized by inducing the expansion of progenitor cells, as well as promoting their differentiation and survival. TNF-*α* induces differentiation and stimulates the proliferation of hematopoietic CD34^+^ cells. IL-4, in turn, inhibits the formation of macrophage colonies [10]. Monocyte-derived dendritic cells can be activated with CD40L or TNF-*α*, since the cells have already been differentiated [8].

A number of alterations regarding surface proteins are observed during differentiation. Monocytes gradually lose CD14 expression yet start to express molecules of CD1 family [8, 11]. When activated, they increase the expression of costimulatory molecules, such as CD80, CD86, and CD40, and express high levels of CD83 and MHC class II [12, 13]. The expression of chemokine receptors also changes during their maturation. Immature dendritic cells express receptors to inflammatory chemokines, such as CCR1, CCR2, CCR5, CCR6, and CXCR1, which facilitate their targeting of the site of inflammation. During activation, CCR6 expression is downregulated, while CCR7 and CXCR4 are upregulated, allowing the movement of cells toward lymph nodes [14–16]. In parallel to these changes, cells modify their activity during development. Immature dendritic cells are strongly capable of endocytosis of potential antigens through different mechanisms involving a number of pattern recognition receptors, whereas activated cells have this ability diminished, albeit displaying increased allostimulatory activity [17, 18].

## 3. Dendritic Cell Plasticity and Subtypes

Dendritic cells are heterogeneous and dynamic cells. Dendritic cells were first characterized by Steinman and Cohn, who isolated them from the spleen of animals and described their remarkable motility and usual conformational change. They also highlighted the fact that, in contrast to B lymphocytes, dendritic cells were unable to increase significantly their numbers in an immune response [19–21]. Therefore, dendritic cell plasticity was always suspected although only recently this has become evident.

These cells can be classified according to their functional development: dendritic cells that are resident in peripheral nonlymphoid tissues and able to recognize and process antigens are called immature. After capturing antigens, dendritic cells migrate to secondary lymphoid organs and acquire the ability to activate lymphocytes. At this point in development, they are classified as mature or activated dendritic cells [22]. Despite some divergence in the literature, these cells are also classified in accordance to their phenotypic differences. Two groups have been proposed: conventional or classical dendritic cells, a group characterized by an integrin expression (CD11c) and in which Langerhans cells and interstitial dendritic cells are included; plasmacytoid dendritic cells, which are producers of interferon (IFN) type I (*α* and *β*), therefore, mainly involved in viral infection responses [23–26]. Nevertheless, there are controversies especially regarding plasmacytoid dendritic cells, due to the fact that they present morphological and phenotypical characteristics of both lymphocytes and classical dendritic cells. A comparative study between human lymphoid and myeloid progenitor cells showed that both can give rise to classical and plasmacytoid cells [25]. In contrast to classical dendritic cells, human plasmacytoid cells do not express CD11c, express low levels of costimulatory molecules and MHC class II, and express a common marker of B lymphocytes, B220. They also differ in the pattern of toll-like receptors (TLRs) expressed, with plasmacytoid cells expressing more TLR 7 and 9 [1, 26].

Independently from the classification, dendritic cells possess the capacity of switching between tolerogenic and effector/cytotoxic phenotypes. Studies in mice characterized immature dendritic cells as cells prone to develop tolerogenic responses while activated dendritic cells were more efficient at promoting effective responses by T cells. This conclusion was based on the fact that dendritic cells resident in peripheral tissues are generally immature and, under homeostatic situations, they induce anergy or T regulatory cell development [27, 28]. The generalization that involves immature dendritic cells as promoters of tolerance versus activated dendritic cells as inducers of effective immune response was accepted for a long time; however, certain stimuli that promote dendritic cell activation are also capable of inducing tolerance, like microbial-derived products [29]. Another example is the treatment of activated dendritic cells with IFN-*γ* which promotes the expression of indoleamine 2,3-dioxygenase (IDO) leading these cells to acquire tolerogenic properties that could be reverted by the inhibition of IDO [30]. Therefore, dendritic cell activities are not dependent on the activation state and they represent a complex group with multiple functional intermediates as opposed to immature and activated cells [31, 32].

Dendritic cell tolerance to self-antigens and to resident nonpathological microorganisms is as essential as the capacity of being immunogenic when a pathogen is present; thus, their ability to switch from these two phenotypes must be finely regulated.

## 4. Dendritic Cells in the Tumor Microenvironment

In the tumor microenvironment the tolerogenic pathway is increased in relation to the effector pathway. Moreover, this microenvironment is generally suppressive to immune cells, which means that immune functions are often prevented, consequently leading to unresponsiveness. Many cell types are affected by tumor cells contact and their various released products. For instance, CD8^+^ T lymphocytes have their cytotoxicity ability compromised [33], NK cells are impaired [34], and macrophages acquire a M2-like phenotype [35, 36].

Dendritic cells are also strongly susceptible to tumor products that may induce important alterations. Analyzing dendritic cell differentiation from human CD34^+^ progenitor cells, the vascular endothelial growth factor (VEGF) was the first tumor-derived protein described as a suppressor of this process [37]. Moreover, it was shown that serine proteases secreted by prostate tumor cells and gangliosides from various tumors inhibited dendritic cell generation in a manner similar to the development (from CD34^+^ cells) in both, humans and mice [38, 39].

Using a different model, monocyte-induced differentiation toward dendritic cells, Menetrier-Caux and collaborators showed that this process was also modulated by tumor products [40]. IL-6 and macrophage colony-stimulating factor (M-CSF) produced by tumors and macrophages present in the tumor microenvironment suppress dendritic cell differentiation, whereas they stimulate macrophage differentiation through the increase of M-CSF receptor expression in monocytes [40].

As discussed by Zou, in 2005, the concentration of cytokines that favor dendritic cell development and function, like GM-CSF, IL-4, IL-12, and IFN-*γ*, is very low, while factors that suppress dendritic cells, such as IL-6, IL-10, prostaglandin E2 (PGE2), VEGF, and transforming growth factor-beta (TGF-*β*), are found in higher levels [34]. In summary, the imbalance of cytokines found in tumor microenvironment does not favor dendritic cell development.

Clinical studies revealed a correlation between the number of dendritic cells inside the tumor and the survival of patients [41]. The presence of a consistent number of CD1a^+^ cells in the tumor microenvironment is associated with a better prognosis [42, 43]. Moreover, Joo and coworkers showed that monocyte-derived CD1a^+^ dendritic cells, during activation process, induce apoptosis and cell cycle arrest in breast cancer cells via secretion of soluble products [44].

Despite the importance of dendritic cells during an antitumoral response, there is clear evidence pointing to the fact that dendritic cells are strongly suppressed by factors present in the tumor microenvironment. Both blood (systemically) and tumor microenvironment (locally) dendritic cells seem to be functionally compromised, which means that tumor products may affect cells in distant sites and possibly undifferentiated cells in the bone marrow [45]. The presence of activated dendritic cells is rare in tumor areas and it has been demonstrated in ovarian, breast, prostate, and some renal carcinomas. All steps of dendritic cell development, migration, and activity may present significant defects [46–49].

**Figure 1:**
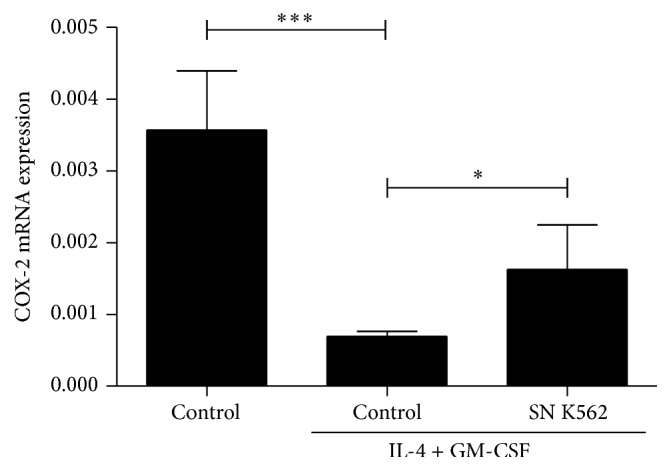
mRNA expression of COX-2 by monocytes differentiated with leukemic cell products. Mononuclear cells were obtained after gradient density from buffy coats of healthy individuals. After 2 h of adhesion, lymphocytes were removed and monocyte cultures were stimulated with IL-4 and GM-CSF (50 ng/mL) to induce dendritic cell differentiation. K562 supernatants (SN K562) were obtained after 3 days of cell culture in RPMI plus 10% of fetal bovine serum (FBS) followed by filtering (0.22 *μ*m). 10% of SN K562 was used since the beginning of monocyte culture until the end (5 days). Afterward, total mRNA was collected using trizol, retrotranscription was performed, and, finally, qPCR was done using COX-2 specific primers. The graph shows mean ± SEM of expression of COX-2 mRNA. ^*∗*^ *p* ≤ 0.05; ^*∗∗∗*^ *p* ≤ 0.001. *n* = 5.

Some workers have tried to reveal the mechanisms involved in tumor-related inhibition of dendritic cells. Among them, Sombroek and collaborators demonstrated that primary tumors (renal carcinoma, cervical cancer, breast cancer and melanoma) negatively modulate the development and activity of dendritic cells through factors regulated by cyclooxygenase-1 (COX-1) and cyclooxygenase-2 (COX-2) [50]. In our hands, investigating a plausible regulation of COX-2 in developing dendritic cells, it was possible to determine that this enzyme was upregulated when cells were under the stimulation of leukemic cell products (Figure 1). Here, once more, we could detect an impairment of dendritic cell development by tumor-derived products.

In another work, Kiertscher et al. showed that monocytes CD14^+^ respond to products present in tumor cell cultures by increasing the expression of antigen-presenting cells surface receptors and increasing the translocation of nuclear factors [51]. However, despite having activated dendritic cells characteristics, these cells lose their ability to secrete IL-12, do not acquire allostimulatory capacity, and rapidly undergo apoptosis [51]. Furthermore, it was shown that cervical adenocarcinoma cells affect the generation of dendritic cells which become incapable of producing IL-12. This has been attributed to the production of IL-10 by tumor cells and consequently to a less expression of CD40 by dendritic cells [52]. Blocking VEGF was the approach used by Osada and coworkers in a study with lung, breast, and colon carcinoma patient cells. They were able to show that anti-VEGF treatment increases cell ability of activating and promoting lymphocyte proliferation [53].

Most studies have been performed with products obtained from solid tumors. In 2010, our group showed that soluble products released by leukemic cells inhibited dendritic cell differentiation through the induction of IL-1*β* and this effect could be partially reversed when IL-1*β* was neutralized in culture [54]. Because we knew the importance of IL-1*β* to tumor progression and metastasis [55], we decided to study the effect of this cytokine along with tumor products on dendritic cell differentiation. Stimuli resulting from tumor products or from IL-1*β* produced different results regarding the appearance of macrophage markers in these cells, with leukemic cell products increasing CD68 and CD16 expression, a feature not observed with IL-1*β*. On the other hand, similarities involving a suppressive phenotype were demonstrated with both kinds of stimuli [56].

In addition to preventing dendritic cell generation and function and stimulating tolerogenic pathways, tumors can induce specific cell phenotypes, such as regulatory dendritic cells and myeloid-derived suppressor cells (MDSCs). Tumor products may induce immature dendritic cells conversion into regulatory dendritic cells, which promote T regulatory cell activation and produce TGF-*β* [32]. In contrast, MDSCs were described as a more undifferentiated population and were characterized in humans by the expression of CD33 [57]. Recent studies showed that the appearance of both phenotypes is directly related to tumor progression and metastasis establishment [32].

Tumors not only represent a pathological condition that is associated with cellular changes, but also represent an organized system of communication between different cells. Diverse and numerous cell types communicate and collaborate in order to establish a favorable environment for the development of cancer. One of the forms of communication used by normal cells, but amplified in neoplastic cells, is the secretion of microvesicles [58]. Microparticles or microvesicles are plasma membrane fragments released by almost all cell types when subjected to stress conditions, including apoptosis. For a long time they were considered only cellular debris. However, more recently it has been shown* in vitro* that microvesicles may reflect cell activation and,* in vivo*, tissue degeneration in various pathophysiological conditions [59]. These structures can vary widely in size (the diameter varies within 0.1–1 *μ*m) and also with regard to their composition [60].

**Figure 2:**
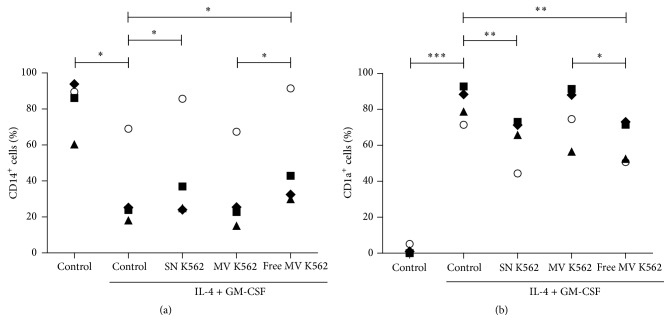
Leukemic cell microvesicles influence on dendritic cell differentiation. Mononuclear cells were obtained after gradient density from buffy coats of healthy individuals. After 2 h of adhesion, lymphocytes were removed and monocyte cultures were stimulated with IL-4 and GM-CSF (50 ng/mL) to induce dendritic cell differentiation. K562 supernatants (SN K562) were obtained after 3 days of cell culture in RPMI plus 10% of fetal bovine serum (FBS) followed by filtering (0.22 *μ*m). Part of the tumor supernatant was centrifuged twice at 100000 g for 1 h to purify microvesicles. After this process, two supernatants were obtained: K562 supernatant only with microvesicles resuspended in the same original volume of medium (MV K562) and K562 supernatant with all the other products except microvesicles (free MV K562). Supernatants were added (10% of final volume) at the beginning of monocyte culture and remained until the end (5 days). Afterward, cells were stained with anti-CD14 FITC and anti-CD1a PE and data were acquired in a FACS Calibur. Graphs show the percentage of CD14^+^ (a) and CD1a^+^ (b) cells. Each individual analyzed in different conditions is represented by one symbol. ^*∗*^ *p* ≤ 0.05; ^*∗∗*^ *p* ≤ 0.01; ^*∗∗∗*^ *p* ≤ 0.001. *n* = 4.

The association of microvesicles with tumors is deduced by the fact that they are secreted in larger amounts by tumor cells and it explains why they circulate at higher levels in the peripheral blood of cancer patients [61–63].* In vitro* studies demonstrated that microvesicles accumulate in tumor cell cultures stimulated or not [64]. Some tumor microvesicles inhibitory effects on immune cells have been described. Baj-Krzyworzeka and collaborators showed that tumor microvesicles interact with monocytes by changing their phenotype [65]. Furthermore, effects like the blockage of proliferation, cytotoxic activity, and the induction of apoptosis in lymphocytes have been shown [66–68]. Suppression of MHC class II molecules expression by murine macrophages was also described [69]. These data suggest that microvesicles derived from tumors may have immunosuppressive characteristics. In our experiments, using leukemic cell products during monocyte differentiation into dendritic cells, we failed to associate inhibition of this process with microvesicles (Figure 2). Here, the comparison performed was between microvesicles-free tumor supernatants and supernatants in which only microvesicles were present. It was observed that the effect on blocking the decrease of CD14 and CD1a appearance was restricted to microvesicles-free supernatant, thus eliminating the role of these tumor microvesicles at least on the CD14 and CD1a modulation (Figure 2).

**Figure 3:**
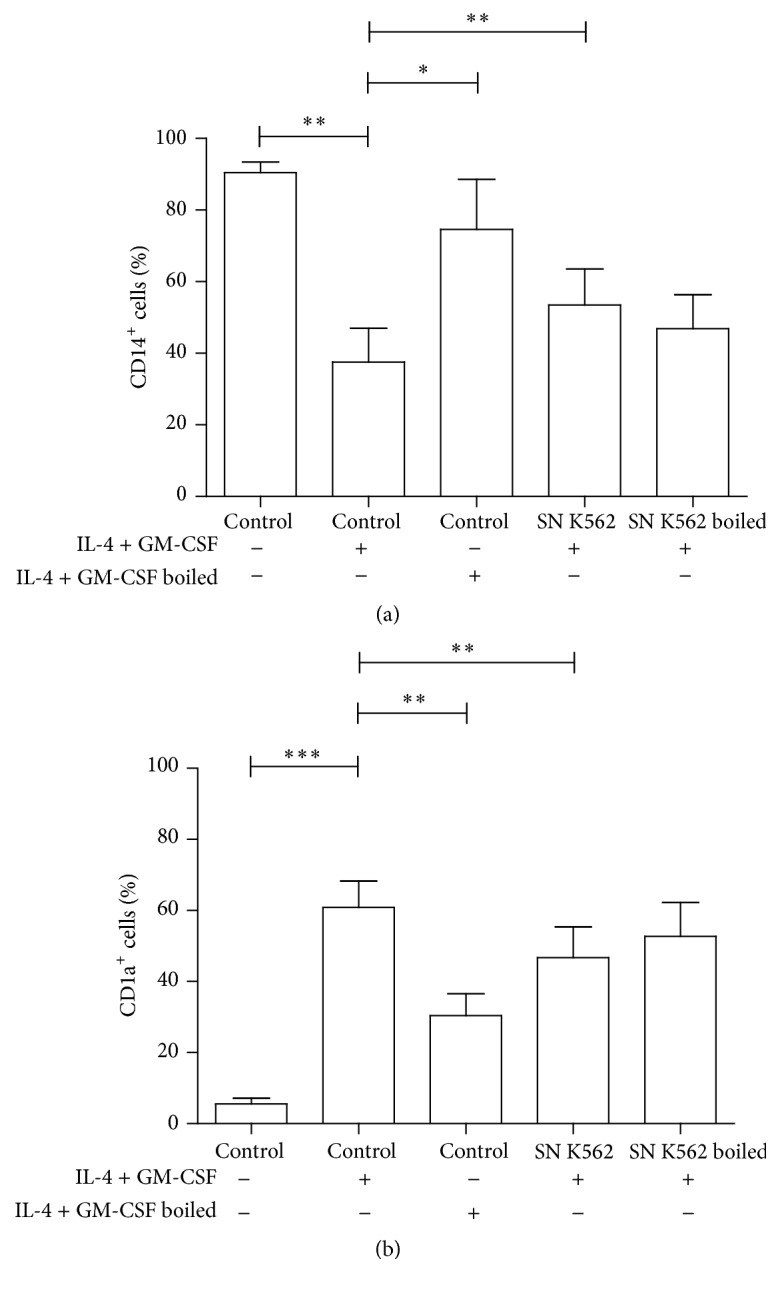
Leukemic cell-derived proteins effect on monocytes differentiating into dendritic cells. Mononuclear cells were obtained after gradient density from buffy coats of healthy individuals. After 2 h of adhesion, lymphocytes were removed and monocyte cultures were stimulated with IL-4 and GM-CSF (50 ng/mL) to induce dendritic cell differentiation. K562 supernatants (SN K562) were obtained after 3 days of cell culture in RPMI plus 10% of fetal bovine serum (FBS) followed by filtering (0.22 *μ*m). Part of tumor supernatant and part of IL-4 and GM-CSF were boiled for 10 minutes at 100°C in order to denature proteins. After cooling, some cells were incubated with these supernatants or cytokines at the same concentration described above for 5 days. Anti-CD14 FITC and anti-CD1a PE were used to stain the cells and data were assessed by a FACS Calibur. Graphs show mean ± SEM of the percentage of CD14^+^ (a) and CD1a^+^ (b) cells. ^*∗*^ *p* ≤ 0.05; ^*∗∗*^ *p* ≤ 0.01; ^*∗∗∗*^ *p* ≤ 0.001. *n* ≥ 5.

Furthermore, experiments were performed to verify whether tumor cell products responsible for the modulation obtained were of a proteic nature. To that end, the tumor supernatant was compared to a supernatant that was boiled at 100°C. In some individuals analyzed, a small reversion of the effect on CD14 and CD1a expression was observed when supernatants were heated, indicating the involvement of any product with a proteic nature. However, residual effects still persisted (Figure 3). Moreover, this was not seen in all the experiments carried out when IL-4 and GM-CSF were heated (used as control). They were still able to induce partial differentiation (Figure 3). Therefore, further investigation is required to better identify the nature of leukemic cell products responsible for dendritic cell generation impairment.

## 5. Dendritic Cell Role in Allograft Rejection

Transplantation is a therapy used for the majority of bone marrow-derived cancers and for many metabolic disorders or failure of diverse organs. Serious complications may occur in transplantation. Bone marrow transplanted patients might suffer from graft-versus-host disease. On the other hand, solid organ transplanted patients might suffer from graft failure. In both situations the risk of infection is a serious problem. Dealing with these issues, it is of great relevance for the understanding of immune mechanisms able to control effective and tolerogenic responses. Contrary to what has been done for immunotherapy against cancer, proposals to avoid transplant rejection are geared towards increasing the ratio tolerance/effective response.

In a model of renal transplantation, an increased number of monocyte-derived dendritic cells in mature stage were found. Moreover this dendritic cell subset was more frequent than the classical dendritic cells that are usually present in major numbers in the kidney. Therefore, a consistent infiltration of monocytes was proposed by Zuidwijk et al. [70]. Another subset of dendritic cells seems to be related to kidney graft rejection. Although plasmacytoid dendritic cells have been described as cells closely associated with tolerance, studies with renal transplantation acute rejection showed that the presence of this subset is not rare. Thus, it could be correlated to inflammation as these plasmacytoid dendritic cells are important producers of IFN [71, 72].

Graft-resident dendritic cells seem to have a protective activity whereas infiltrating dendritic cells might act as proinflammatory cells. This idea was reinforced by experiments with animals in which the progressive infiltration of dendritic cells and CD4^+^ T lymphocytes increased effector responses against the graft in a kidney model of transplantation [73].

Interestingly, liver transplantation is better accepted in patients and its grafting may be able to enhance the acceptance of other organs, like heart and skin allografts. Therefore, it was proposed that the liver microenvironment favors immune tolerance not only locally, but also systematically [74]. Most dendritic cells that reside in the liver display a typical immature phenotype with low expression of costimulatory molecules. Furthermore, dendritic cell-mediated tolerance in liver can be explained by the expression of programmed death ligand 1 (PD-L1) by these cells as well as cytotoxic T lymphocyte antigen 4 (CTLA-4) that was found to be highly expressed by T lymphocytes in the organ [75, 76]. Even though liver dendritic cells may produce IL-12, the IL-10 level is increased in this organ compared to others and, in part, it may explain the tolerogenic behavior of liver-resident dendritic cells [74, 77].

Certain subsets of dendritic cells have been related to heart graft rejection. A role for CD11c^+^ dendritic cells in heart graft rejection has been demonstrated by Oberhuber et al. [78]. Making use of a murine model, they showed that intragraft CD11c^+^ cells enhance CD4^+^ and CD8^+^ T cells responses and the production of cytokines, such as IL-17A. Conversely, depletion of this population of dendritic cells or blocking of IL-17A is able to delay the rejection of transplanted heart [78].

The generation of regulatory dendritic cells in this circumstance could be interesting. Murine regulatory dendritic cells that lack the expression of costimulatory proteins were induced* in vitro* and injected in mice 7 days before heart transplantation. Results demonstrated longer graft survival even in mice that were not treated with immunosuppressive drugs [79, 80].

One of the most studied types of transplantation is the skin graft due to the high index of rejection. Dermal dendritic cells and Langerhans cells (present in epidermis) are the main subsets of dendritic cells in normal skin. In skin transplantation, both populations of dendritic cells migrate out of the graft in the direction of draining lymph nodes where they present donor antigens to T recipient cells. In this context, T cells from the recipient may recognize antigens from the donor dendritic cells (direct allorecognition) and also donor antigens presented by recipient dendritic cell (indirect allorecognition) [81, 82]. A third way of T cell activation is the recognition of donor MHC previous transferred to recipient dendritic cells (semidirect recognition) [83]. Thus, the activation of T cells occurs in a very efficient way in skin transplantation and it explains why this kind of graft is frequently rejected.

Solid organs transplantations require immunosuppressive therapies in order to ensure graft acceptance. However, bone marrow transplantation is unique because in this case the recipient immune system is destroyed and replaced by the donor system. Therefore, the new immune system is capable of rejecting not the graft but all the other organs. Immunosuppressive therapies are also required for this kind of transplantation, even though for different reasons [84].

The induction of peripheral tolerance is the main goal to avoid graft rejections and manipulating dendritic cells to acquire a tolerogenic activity is one of the mechanisms to reach this objective. Carreño and collaborators suggested the blockage of NF-*κ*B as a way to induce tolerogenic characteristics in dendritic cells [85]. Recently, some immunosuppressive agents, which have been used to treat autoimmune disease and to avoid allograft rejection, were described as inductors of tolerance in dendritic cells [86].

## 6. Final Considerations

**Figure 4:**
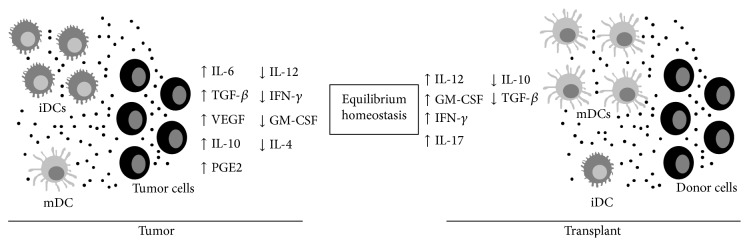
Schematic illustration showing the differences between tumor and transplants microenvironments. Immature dendritic cells (iDCs) are present in higher number in tumor microenvironments, while activated dendritic cells (mDCs) can be more easily found in transplanted organs. In both cases, tumor cells or donor cells and dendritic cells are in constant communication through cytokines and other factors secretion (represented by small black circles). In the tumor microenvironment, factors that are generally suppressive to dendritic cells are present in big amounts. On the other hand, in transplants, proinflammatory cytokines which exacerbate dendritic cells function are increased.

The plasticity of dendritic cells is evident and it is essential to keep the homeostasis of normal individuals. However, due to the capacity of these cells to easily switch phenotypes according to microenvironmental signals, this plasticity might be harmful when disorders such as cancer occur or when an allotransplantation is required (Figure 4). Nevertheless, lessons learned on how these situations may affect dendritic cells might help to understand how to handle the modulation connected to modifications in the microenvironment resulting from diverse pathologies. Here we discussed two paradoxical situations in which the balance of tolerance/effective immune response was considered especially looking at dendritic cell phenotype and function.
